# Anaplastic Lymphoma Kinase (ALK) Fusion as a Resistance Mechanism to Epidermal Growth Factor Receptor (EGFR) Tyrosine Kinase Inhibitors With Complete Response After Osimertinib and Alectinib: A Case Report and Literature Review

**DOI:** 10.7759/cureus.92202

**Published:** 2025-09-13

**Authors:** Ben Man Fei Cheung, Aya El Helali, Dennis Kwok Chuen Leung

**Affiliations:** 1 Department of Clinical Oncology, Queen Mary Hospital, Hong Kong, HKG; 2 Department of Clinical Oncology, The University of Hong Kong, Hong Kong, HKG

**Keywords:** alectinib, alk fusion, egfr mutation, eml4-alk, non-small-cell lung cancer, osimertinib

## Abstract

Acquired resistance to epidermal growth factor receptor (EGFR) tyrosine kinase inhibitors (TKIs) remains a significant challenge in the management of EGFR-mutant non-small-cell lung cancer (NSCLC). While EGFR T790M mutation and MET amplification are well-established resistance mechanisms, the development of secondary oncogenic fusions such as anaplastic lymphoma kinase (ALK) rearrangements is rare but clinically actionable.

We report a 62-year-old male with EGFR exon 19 deletion-positive NSCLC who initially responded to osimertinib but developed progressive brain metastasis after 13 months of treatment. Next-generation sequencing of the brain metastasis revealed an acquired EML4::ALK fusion while maintaining the original EGFR exon 19 deletion. The patient was treated with a combination of osimertinib and alectinib, achieving a complete response that has been sustained for 12 months without significant toxicity.

Literature review identified 16 reported cases of ALK fusion as a resistance mechanism to EGFR TKIs. The median time to development of ALK fusion resistance was 16 months (range: 6-34 months). Most patients (75%) developed secondary ALK fusion after osimertinib exposure. EML4::ALK was the most common fusion variant (68.8%), though this population showed enrichment for uncommon ALK fusion partners compared to de novo ALK-positive NSCLC. Central nervous system involvement was common (33% of cases). Combination EGFR and ALK TKI therapy demonstrated an objective response rate of 66.7% with good tolerability.

Secondary ALK fusion represents a rare but important resistance mechanism to EGFR TKIs that can be effectively treated with combination targeted therapy. This is particularly clinically relevant as treatment with dual EGFR and ALK TKI has a high response rate with low toxicity in an otherwise treatment-refractory population with poor survival. Comprehensive molecular profiling at progression is essential for identifying this actionable resistance mechanism.

## Introduction

The emergence of acquired resistance remains a major challenge in the management of epidermal growth factor receptor (EGFR)-mutant non-small-cell lung cancer (NSCLC) treated with tyrosine kinase inhibitors (TKIs). While EGFR T790M and MET amplification are well-established resistance mechanisms, the development of secondary oncogenic fusions, such as anaplastic lymphoma kinase (ALK) rearrangements, is rare but clinically actionable [1].

EGFR-mutant NSCLC represents approximately 10-15% of lung adenocarcinomas in Western populations and up to 50% in East Asian populations [2]. Despite significant advances with third-generation EGFR TKIs like osimertinib, which has demonstrated superior efficacy with a median progression-free survival of 18.9 months in first-line treatment [3], acquired resistance remains inevitable. Secondary ALK fusions occur in approximately 1-3% of patients with acquired resistance to EGFR TKIs [4]. Unlike other resistance mechanisms that may require chemotherapy, ALK fusions represent actionable targets that can be effectively treated with approved ALK inhibitors [5]. However, the optimal management strategy for patients with concurrent EGFR mutations and secondary ALK fusions remains unclear, particularly regarding combination targeted therapy approaches [6].

This case report describes a patient with EGFR-mutated NSCLC who developed an acquired EML4::ALK fusion after progression on osimertinib, and achieved a durable complete response with combination osimertinib and alectinib. Existing literature was also reviewed to shed light on this rare but important resistance mechanism.

## Case presentation

A 62-year-old male initially presented with weakness and clumsiness. He had a performance status of 0 on presentation. MRI brain revealed a 2.5 cm high frontal contrast-enhancing lesion along the midline, suspicious of brain metastasis. Whole body PET-CT showed a 2.6 cm lung mass over the left lower lobe as well as iliac bone metastasis. Biopsy of the left lower lobe lung mass revealed adenocarcinoma of lung origin. Next-generation sequencing (NGS; Oncomine Precision Assay, Ion Torrent Genexus System, Thermo Fisher Scientific, Waltham, MA) of the tumor demonstrated EGFR exon 19 deletion mutation. ALK was negative by both NGS and immunohistochemistry (IHC) at the time. No other mutations were present.

**Figure 1:**
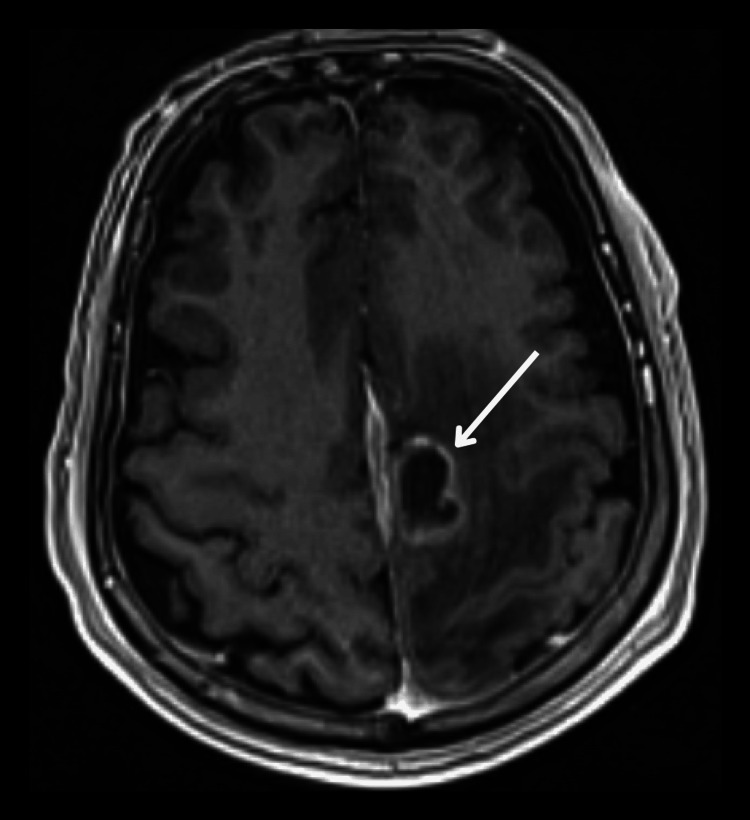
Initial presentation and early progression on osimertinib. Axial T1-weighted contrast-enhanced MRI brain image showing a solitary 2 cm enhancing lesion in the left frontal lobe at the time of disease progression on osimertinib. This lesion was surgically resected and found to harbor both EGFR exon 19 deletion and acquired EML4::ALK fusion. EGFR: epidermal growth factor receptor; ALK: anaplastic lymphoma kinase.

The patient was started on osimertinib (80 mg daily) with a good response. Interval PET-CT and MRI brain demonstrated partial response to osimertinib, with resolution of neurological symptoms. The disease remained under control for one year. At this time, the patient developed new-onset right upper limb weakness and clumsiness. MRI brain demonstrated a solitary 2 cm lesion over the left frontal lobe (Figure 1). PET-CT showed sustained complete response for extracranial disease. Craniotomy with excision of brain metastasis was done. NGS (Oncomine Precision Assay, Ion Torrent Genexus System, Thermo Fisher Scientific) of the brain metastasis was done, which was positive for EGFR exon 19 deletion as well as ALK fusion rearrangement. The specific ALK fusion was EML4::ALK. Orthogonal testing showed that ALK was positive by IHC as well. The EGFR T790M mutation was negative. Postoperative stereotactic radiotherapy was delivered to the surgical cavity at 32.5 Gy over five fractions. In view of the complete resection of all progressive lesions, the patient was continued on osimertinib.

**Figure 2:**
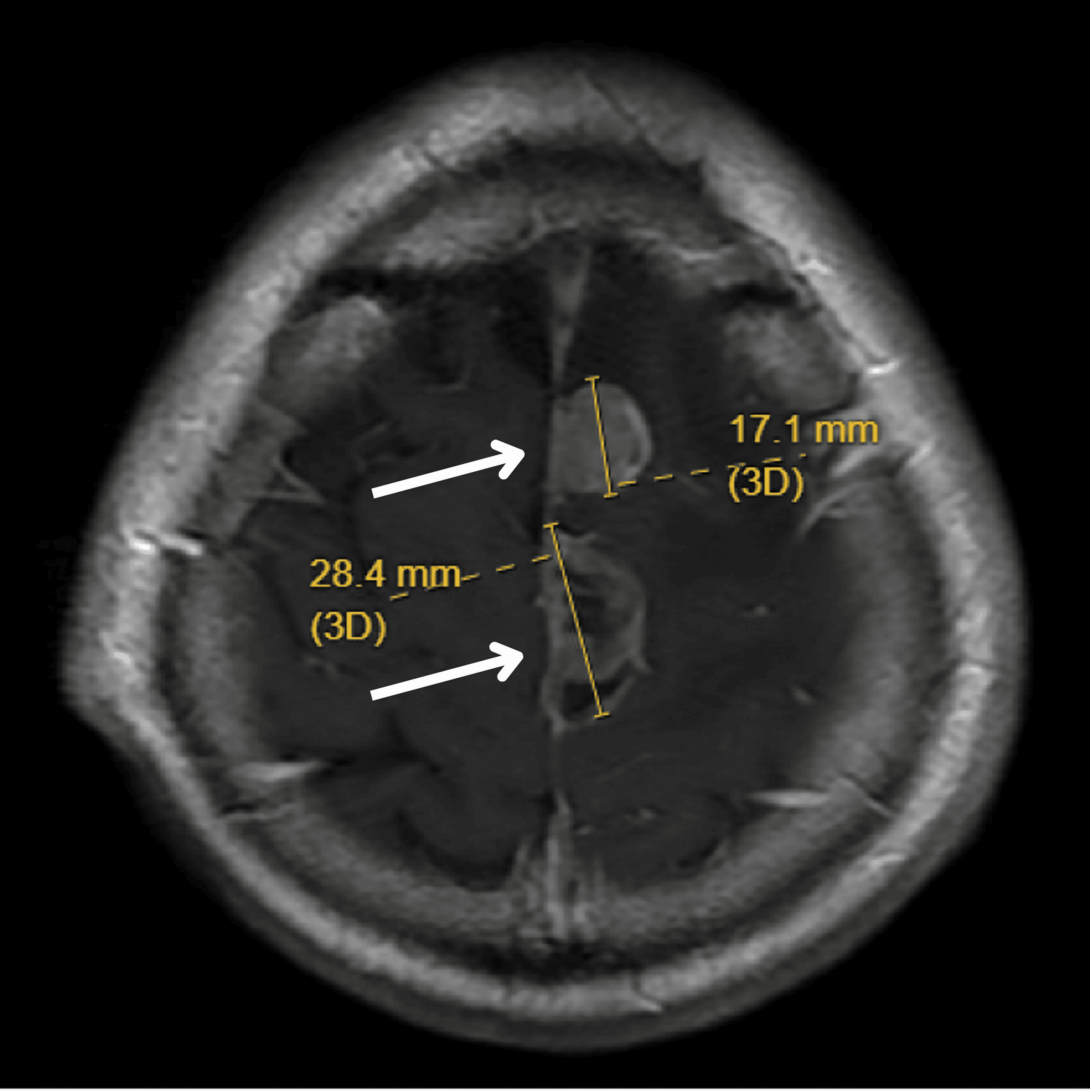
Disease progression on surgical resection and radiotherapy. Axial T1-weighted contrast-enhanced MRI brain image demonstrating new parasagittal dural metastasis, occurring three months after surgical resection and stereotactic radiotherapy. The patient developed status epilepticus at this time and was treated with palliative whole-brain radiotherapy.

However, the patient developed status epilepticus three months later. MRI brain showed a new parasagittal dural metastasis (Figure 2). The patient was treated with palliative whole brain radiotherapy (WBRT) at 20 Gy in five fractions.

**Figure 3:**
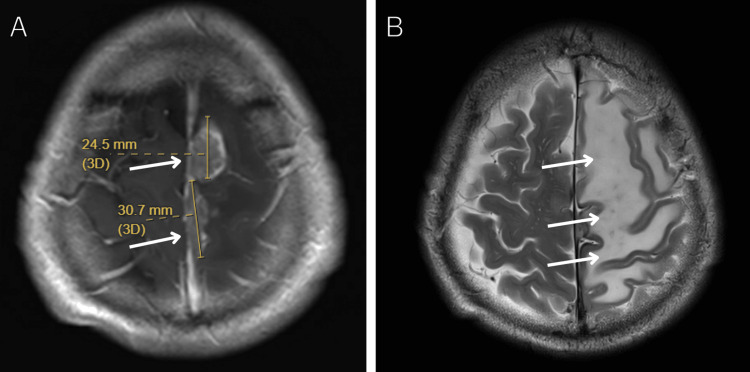
Disease progression on osimertinib monotherapy. (A) Axial T1-weighted contrast-enhanced MRI brain image showing progressive parasagittal lesion after whole-brain radiotherapy while on continued osimertinib monotherapy. Measurements indicate tumor dimensions of 24.5 mm and 30.7 mm. (B) Axial T2-weighted MRI brain image showing significant perilesional edema, demonstrating continued disease progression despite osimertinib treatment.

The patient was continued on osimertinib (80 mg daily) and interval MRI brain four months after WBRT demonstrated progressive parasagittal lesion while extracranial disease was still in remission (Figure 3).

**Figure 4:**
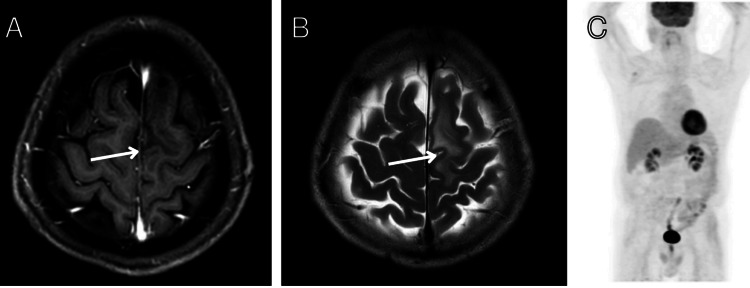
Complete response to combination therapy. (A) Axial T1-weighted contrast-enhanced MRI brain image demonstrating complete response of the dural metastasis four months after initiation of combination osimertinib and alectinib therapy. (B) Axial T2-weighted MRI brain image demonstrating near complete resolution of edema four months after initiation of combination osimertinib and alectinib therapy. (C) Whole body PET-CT demonstrating complete extracranial response four months after initiation of combination osimertinib and alectinib therapy, showing no evidence of metabolically active disease.

ALK fusion was hence deemed to be the resistance mechanism to osimertinib. The patient was started on a combination of osimertinib (80 mg daily) and alectinib (600 mg twice daily). Four months after starting combination EGFR and ALK inhibitor, interval MRI brain demonstrated complete response of dural metastasis with resolution of all neurological symptoms (Figures 4A, 4B). The patient is on osimertinib with alectinib to date, with a complete response for one year (Figure 4C). The patient had regular follow-ups every month for safety monitoring. The patient did not experience any significant toxicity from treatment.

**Figure 5:**
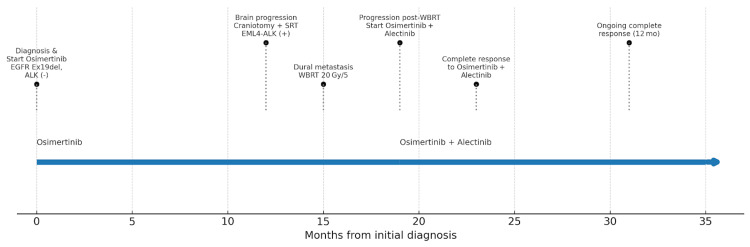
Treatment timeline. Timeline illustrating the patient's treatment course from initial diagnosis through combination targeted therapy. The timeline shows initial presentation with brain and extracranial metastases, response to osimertinib, development of brain-specific progression with acquired ALK fusion, and subsequent complete response to combination osimertinib and alectinib therapy. Key molecular findings and treatment interventions are highlighted at relevant time points. EGFR: epidermal growth factor receptor; ALK: anaplastic lymphoma kinase; SRT: stereotactic radiotherapy; WBRT: whole brain radiotherapy.

The timeline of treatment is presented in Figure 5.

## Discussion

Resistance mechanism to EGFR inhibitors is heterogeneous [7]. The most commonly described mechanism of resistance to EGFR inhibitors was the T790M mutation [1]. Other documented mutations included the EGFR C797X mutation, MET amplification, and HER2 amplification [8]. Emergence of oncogenic fusion as a resistance mechanism to EGFR TKIs is a rare but documented phenomenon estimated to account for 3-10% of resistance [1]. Amongst patients with secondary oncogenic fusion, ALK was the second most common resistance mechanism, accounting for 23% of the alterations, while BRAF fusion was the most common (32%) [9]. Here, we reviewed all reported cases of ALK fusion as a resistance mechanism to EGFR TKIs in the literature (Table 1).

**Table 1:** Characteristics of patients with ALK fusion as a resistance mechanism to EGFR TKI. ALK: anaplastic lymphoma kinase; CNS: central nervous system; CR: complete response; EGFR: epidermal growth factor receptor; IHC: immunohistochemistry; ILD: interstitial lung disease; N/A: not available; NGS: next-generation sequencing; PR: partial response; SD: stable disease; TKI: tyrosine kinase inhibitor.

Case	First author (year)	Age/sex	EGFR mutation	EGFR-TKI history	ALK fusion	Interval to ALK resistance (months)	Post-resistance regimen	Best response	Grade ≥3 adverse events	Biopsy site	Testing	CNS progression	Extracranial progression site(s)
1	Present case	65/M	Exon 19 del	Osimertinib	EML4-ALK	13	Osimertinib + alectinib	CR	None	Brain	IHC + NGS	Yes	No
2	Schrock et al. (2018), Case 7 [9]	63/F	Exon 19 deletion	Erlotinib → osimertinib	EML4-ALK	16	N/A	-	N/A	N/A	N/A	N/A	N/A
3	Schrock et al. (2018), Case 9 [9]	46/F	L747P	Afatinib	EML4-ALK	21	N/A	N/A	N/A	N/A	N/A	N/A	N/A
4	Schrock et al. (2018), Case 10 [9]	67/F	L858R → T790M	Erlotinib → afatinib → osimertinib	PLEKHA7-ALK	29	Osimertinib + alectinib	PR	N/A	Liquid	NGS	N/A	N/A
5	Schrock et al. (2018), Case 11 [9]	61/M	L858R	Erlotinib → afatinib	EML4-ALK	7.5	N/A	N/A	N/A	N/A	N/A	N/A	N/A
6	Schrock et al. (2018), Case 26 [9]	68/M	Exon 19 deletion	Erlotinib → osimertinib	STRN-ALK	N/A	No	N/A	N/A	N/A	N/A	N/A	N/A
7	Schrock et al. (2018), Case 27 [9]	62/M	Exon 19 deletion	Afatinib	EML4-ALK	N/A	No	N/A	N/A	Liquid	NGS	N/A	N/A
8	Schrock et al. (2018), Case 28 [9]	60/F	L858R	Osimertinib	TFG-ALK	N/A	No	N/A	N/A	Liquid	NGS	N/A	N/A
9	Liang et al. (2016) [10]	46/F	Exon 19 deletion	Erlotinib → chemotherapy + bevacizumab → osimertinib	EML4-ALK	6	Osimertinib + crizotinib → osimertinib + brigatinib	PR	None	Liquid	NGS	No	Liver
10	Offin et al. (2018), Case 1 [11]	57/F	Exon 19 deletion → T790M	Erlotinib → osimertinib + necitumumab	EML4-ALK	23	Osimertinib + crizotinib	PR	None	Lung	IHC + NGS	No	Lung
11	Offin et al. (2018), Case 2 [11]	65/F	Exon 19 deletion → T790M	Erlotinib → osimertinib	EML4-ALK	21	Osimertinib + alectinib	PR	None	Lung	IHC + NGS	No	Lung
12	Zeng et al. (2021) [12]	59/M	L858R → T790M	Gefitinib	EML4-ALK, STRN-ALK	10	Osimertinib + crizotinib	SD	None	N/A	N/A	No	Liver & bone
13	Hou et al. (2021) [13]	60/F	Exon 19 deletion	Gefitinib → osimertinib	EML4-ALK	32	Osimertinib + crizotinib → brigatinib → osimertinib + crizotinib → chemotherapy + bevacizumab + osimertinib + dasatinib → osimertinib + EGFR antibody → chemotherapy → osimertinib + alectinib	SD	Brigatinib-induced ILD	Lung	NGS	No	Lung
14	von Buttlar et al. (2021) [14]	78/F	Exon 19 deletion	Osimertinib	EML4-ALK	34	Osimertinib + alectinib	PR	None	Lung	NGS	Yes	Lung
15	Yin et al. (2022) [15]	60/F	Exon 19 deletion + T790M (de novo)	Osimertinib	DCTN1-ALK	16	Alectinib	PR	None	Liquid	NGS	Yes	Lung, pleural effusion
16	Portugal et al. (2023) [16]	42/F	L858R	Osimertinib	EML4-ALK	10	Osimertinib + alectinib	SD	None	Lung	NGS	No	Lung, adrenal

A total of 16 cases of ALK fusion resistance to EGFR TKIs were recorded. Median time to development of ALK fusion resistance was 16 months (range: 6-34 months). The most common primary EGFR mutation was exon 19 deletion (56%; n = 9), while the L858R mutation consisted of 31% of the reported cases (n = 5). Most patients developed secondary ALK fusion after exposure to osimertinib (n = 12). However, three patients developed secondary ALK fusion after afatinib, while one patient developed ALK fusion after gefitinib. This indicated that secondary ALK fusion is not a resistance mechanism specific to treatment with osimertinib but secondary to EGFR TKIs treatment in general.

Most of the secondary ALK fusion gene was EML4::ALK fusion (68.8%; n = 11). However, there were multiple reported rare ALK fusion partners as well, including STRN, DCTN1, PLEKHA7, and TFG. Though EML4::ALK remained the most prevalent fusion type, this population is still highly enriched for uncommon ALK fusions (31.2%). For comparison, EML4 was documented to account for over 95% of fusion variants in de novo ALK mutant NSCLC [17].

Central nervous system (CNS) involvement was common. A total of 33% (n = 3) of all cases with reported disease site (n = 9) had CNS involvement. Secondary ALK mutant NSCLC showed a high propensity for CNS metastasis consistent with historical rates of CNS metastasis in de novo ALK mutant NSCLC [18]. This highlights the need for CNS-penetrating TKIs such as lorlatinib or alectinib, as well as CNS-directed local therapies.

ALK-directed TKI was effective in the treatment of secondary ALK fusion. For patients on combination EGFR and ALK-directed TKI, the objective response rate was 66.7% (stable disease = 3; partial response = 5; complete response = 1; N = 9). The most common regimen was osimertinib plus alectinib (n = 5), followed by osimertinib plus crizotinib (n = 4). Combination of EGFR inhibitor with ALK inhibitor was well tolerated without any grade three or beyond adverse events. The only grade three adverse event documented was brigatinib-induced interstitial lung disease whilst on single-agent brigatinib. The patient later tolerated osimertinib plus crizotinib without high-grade toxicities.

The above review of the literature demonstrated the effectiveness and safety of combination EGFR and ALK-directed TKIs in this rare acquired resistance [19]. Importantly, the combination of EGFR and ALK TKIs was effective in rare ALK fusion alterations as well. Given the propensity for CNS spread in EGFR and ALK co-altered tumors, osimertinib plus alectinib or lorlatinib would be preferable [18].

We acknowledged the following limitations for the current study. Firstly, generalizability is limited by the retrospective nature of the case report. Prospective registries may aid generalizability in the future. Secondly, there was a lack of serial circulating DNA monitoring. By monitoring circulating DNA, the clonal evolution of the present case can be studied. Finally, the literature review of case reports and case series was limited to pooling and comparison due to incomplete reporting of outcomes. Prospective basket trials on combination treatment with multiple TKIs may provide evidence on the efficacy of dual EGFR-ALK blockade.

## Conclusions

Secondary ALK fusion represents a rare but clinically significant resistance mechanism for NSCLC patients treated with EGFR TKIs, occurring in a small number of resistance cases with a median time to development of 16 months. Comprehensive molecular profiling at the time of disease progression is essential for identifying this patient subgroup, as early detection enables prompt initiation of appropriate targeted therapy. Orthogonal validation with immunohistochemistry remains important when tissue is available to confirm NGS findings. This resistance mechanism is not specific to any particular EGFR TKI and has been observed across different generations of EGFR inhibitors. The molecular landscape of secondary ALK fusions differs from de novo ALK-positive NSCLC, with enrichment for uncommon fusion partners beyond the typical EML4-ALK rearrangement. CNS involvement is common at 33% in this patient population, emphasizing the importance of using CNS-penetrant targeted therapies. Combination therapy with EGFR and ALK inhibitors demonstrates promising efficacy with an objective response rate of 66.7% and an acceptable tolerability profile, making it a viable treatment option for this rare but actionable resistance mechanism.
